# An applied methodology for stakeholder identification in transdisciplinary research

**DOI:** 10.1007/s11625-016-0385-1

**Published:** 2016-07-26

**Authors:** Julia Leventon, Luuk Fleskens, Heleen Claringbould, Gudrun Schwilch, Rudi Hessel

**Affiliations:** 1 Faculty of Sustainability, Leuphana University, Scharnhorststr. 1, 21335 Lüneburg, Germany; 2 Sustainability Research Institute, University of Leeds, Woodhouse Lane, Leeds, LS2 9JT UK; 3 Soil Physics and Land Management Group, Wageningen University, Droevendaalsesteeg 4, 6708 PB Wageningen, The Netherlands; 4 Consult and Research on Participation and Gender in Environmental issues (Corepage), Buys Ballotstraat 35, 3572 ZT Utrecht, The Netherlands; 5 Centre for Development and Environment CDE, University of Bern, Hallerstrasse 10, 3012 Bern, Switzerland; 6 Team Soil, Water and Land Use, Alterra, Wageningen UR, P.O. Box 47, 6700 AA Wageningen, The Netherlands

**Keywords:** Sustainability, Participation, Soil degradation, Interdisciplinarity

## Abstract

In this paper we present a novel methodology for identifying stakeholders for the purpose of engaging with them in transdisciplinary, sustainability research projects. In transdisciplinary research, it is important to identify a range of stakeholders prior to the problem-focussed stages of research. Early engagement with diverse stakeholders creates space for them to influence the research process, including problem definition, from the start. However, current stakeholder analysis approaches ignore this initial identification process, or position it within the subsequent content-focussed stages of research. Our methodology was designed as part of a research project into a range of soil threats in seventeen case study locations throughout Europe. Our methodology was designed to be systematic across all sites. It is based on a snowball sampling approach that can be implemented by researchers with no prior experience of stakeholder research, and without requiring significant financial or time resources. It therefore fosters transdisciplinarity by empowering physical scientists to identify stakeholders and understand their roles. We describe the design process and outcomes, and consider their applicability to other research projects. Our methodology therefore consists of a two-phase process of design and implementation of an identification questionnaire. By explicitly including a design phase into the process, it is possible to tailor our methodology to other research projects.

**Electronic supplementary material:**

The online version of this article (doi:10.1007/s11625-016-0385-1) contains supplementary material, which is available to authorized users.

## Introduction

Early identification of stakeholders around a natural resource is critical for meaningful transdisciplinary research into the management of that resource. A stakeholder is any actor that can affect, or can be affected by, a decision or action (after Freeman 1984). Researchers in natural resource management consistently find that stakeholders should be included in solution-finding in order to facilitate negotiation and mutual learning; reduce conflict; and increase support and actor buy-in for decisions made (e.g. Grimble and Wellard 1997; Ravnborg and Westermann 2002; Dougill et al. 2006). Transdisciplinary research approaches build on such rationale by bringing together stakeholders to integrate their different forms of knowledge and ideas in solution-oriented, socially robust research (Lang et al. 2012; Bracken et al. 2014; Hurni and Wiesmann 2014). In an ideal transdisciplinary research project, integration between science and society should occur to shape research agendas, produce knowledge, and incorporate such knowledge into social and scientific practice (Jahn et al. 2012). Stakeholders could be engaged in transdisciplinary research for three main reasons: (1) normative, in order to represent a democratic ideal by focussing on the process of inclusion; (2) substantive, to harness knowledge and risk perceptions from stakeholders in order to improve outcomes; and (3) instrumental, to increase the legitimacy of pre-defined decisions and therefore increase effectiveness [(Fiorino 1990, and as expanded on by Stirling (2008)].

Prior to undertaking any form of transdisciplinary research, researchers need to understand the stakeholder environment, in order that they can consider whom to include and how that impacts upon achieving the purpose. Stirling (2008) highlights that including only experts ‘closes down’ the participation space and narrows the scope for appraisal. This may be appropriate for some kinds of decisions and actions (Mathie and Greene 1997). However, ‘opening up’ to include non-experts widens discourses, which should be a precondition of appraising decisions and shaping agendas (Stirling 2008). Key to such opening up is to include a diversity of stakeholder perspectives in order that a broad range of ideas and opinions are highlighted, and can be contested and discussed by participants (Cuppen 2012). In particular, marginalised actors must be engaged in a way that allows real influence in the decision-making process (Wester et al. 2003). However, a key challenge in transdisciplinary research lies in knowing who the stakeholders are in the first place, and thus identifying the population from which the sample of stakeholders can be drawn. This requires moving beyond pre- and narrowly defined networks to ensure participation from the ‘right’ stakeholders at appropriate times in the research to achieve these ideals (Stauffacher et al. 2008; Lang et al. 2012). Thus a necessary precursor to transdisciplinary research is a stakeholder identification phase that provides a baseline understanding of stakeholders, including who they are and what their roles are. Such a baseline allows researchers to consider who the stakeholders are prior to considering who to engage with, and how to do so, in the transdisciplinary research.

In this paper, we outline a systematic (yet flexible) methodology for stakeholder identification. The methodology allows for early identification of stakeholders. Once identified, researchers can consider which stakeholders they need to engage with and how, thus allowing productive engagement of stakeholders in transdisciplinary research projects. We formulated our approach for use across diverse case study sites in the transdisciplinary research project RECARE. The RECARE project examines a range of soil threats across European countries. In total, there are 17 different case studies, summarised with location and soil threat in Table 1. The project is establishing stakeholder platforms in each case study location to foster a joint learning environment for stakeholders and researchers, with the intention of producing solutions-orientated research (Schwilch et al. 2012). However, before solutions can be co-produced, deliberations with stakeholders about what the problem is should be part of the process. Through a structured process using a series of stakeholder workshops and other transdisciplinary elements, the causes, impacts, problems and possible solutions to soil threats are explored and sustainable land management practices identified and tested with land users (see Hessel et al. 2014 for details). Thus, the RECARE project seeks substantive stakeholder engagement in order to bring together different types of knowledge to produce better soil management outcomes. Because of its strong reliance on stakeholder engagement, and the wide variety in stakeholders anticipated between the case studies, the project provided an ideal opportunity to develop a simple yet structured identification procedure.

**Table 1:** Case study sites in the RECARE project

Case study	Primary soil threat	Location	Country
1	Erosion	Frienisberg	Switzerland
2	Erosion	Caramulo	Portugal
3	Erosion	Peristerona watershed	Cyprus
4	Salinization	Timbaki, Crete	Greece
5	Compaction	Aarslev	Denmark
6	Soil sealing	Poznan and Wroclaw	Poland
7	Desertification	Canyoles River Basin	Spain
8	Desertification	Gunnarsholt	Iceland
9	Floods and Landslides	Vansio-Hobol Catchment	Norway
10	Floods and Landslides	Mjava Catchment	Slovak Republic
11	Loss of organic matter	Veenweidegebied	The Netherlands
12	Loss of organic matter	Broddbo	Sweden
13	Loss of organic matter	Olden Eibergen	The Netherlands
14	Loss of organic matter	Veneto Region	Italy
15	Contamination	Guadiamar	Spain
16	Contamination	Copsa Mica	Romania
17	Loss of soil biodiversity	Isle of Purbeck	United Kingdom

We share our approach for use in future research because a transparent stakeholder identification process to precede and inform transdisciplinary research is largely absent from the literature. Often descriptions in academic articles and project reports of how stakeholders were identified are opaque. Therefore researchers new to a topic, location, or indeed to transdisciplinary research, are left without clear examples and tools to start their research. Several excellent review papers have provided typologies to understand and analyse stakeholders around natural resource management (e.g. Reed 2008; Prell et al. 2009; Reed et al. 2009). However, there is little information on how to identify a population of stakeholders in the first place. Practitioner-oriented guidelines are more helpful; for example guidelines produced by the Caribbean Natural Resources Institute (Renard 2004) include simple steps and questions for a person to identify stakeholders (p.7). However, these still place significant emphasis on the researcher’s knowledge, and are more vague on *how* (and with whom) to answer identification questions. Because transdisciplinary research encourages scientists to engage with stakeholders in generating knowledge and solutions, we seek to fill this gap and provide a structured and useable tool for stakeholder identification.

Our approach to stakeholder identification is novel because it bridges between two emerging approaches to stakeholder identification: collective identification and researcher immersion. Under collective identification, transdisciplinary researchers incorporate identification into the first research session (usually a workshop) with stakeholders (see, e.g., Dougill et al. 2006). In this case, a preliminary group are identified by the researchers using their prior knowledge. Then further stakeholders are identified by the researchers and these stakeholders while knowledge is already being created, meaning that some stakeholders are not engaged from the very beginning of the project. Conversely, under researcher immersion, researchers (e.g., Dyer et al. 2013) focus on identification prior to the first data-generation exercise by immersion in a research problem. In this case, the researchers perform initial desk-based research to identify stakeholders, and then extend this knowledge through exploratory or pilot studies in a case study area. The researchers then organise a stakeholder engagement event, where the researchers and stakeholders generate knowledge. However, this approach is done by focussing on one (or a few) case study, with long lead-in times, and substantial resources and social science skills committed to identifying stakeholders (see, for example Ravnborg and Westermann 2002; Leventon and Antypas 2012). By bridging these two approaches, we are able to build on researcher knowledge without requiring long lead-in times or large resources, in order to identify stakeholders prior to trying to engage with them in the research project.

Our stakeholder identification methodology comprises a conceptually informed questionnaire that is refined to the specific research (and researcher) context via a preliminary design phase. In presenting this methodology, we first outline the theoretical requirements for stakeholder identification for transdisciplinary research. This provides a conceptual framing for our methodology. We supplement this by considering the practical design challenges presented by undertaking identification across a range of case study areas and contexts. We then present the practical development of the stakeholder identification questionnaire. Here, we highlight how we designed the questionnaire to match the conceptual framing, and the practical demands of the RECARE project. The proceeding section then describes the identification process, including the questionnaire and supporting materials, and practical implementation steps. This section includes a critical reflection on the implementation process as conducted in RECARE. Our discussion section continues by reflecting on the extent to which our questionnaire met its conceptual goals, and provided an in-depth understanding of the stakeholder baseline in each case study, such that case studies could consider stakeholders for possible inclusion in the transdisciplinary research stages of the project. We therefore highlight a number of improvements or modifications that could be made. We discuss how the methodology can be applied to other transdisciplinary research projects. In this way, we provide a transferable approach to identify stakeholders, and understand the stakeholder environment for sustainability research, in order that informed selection and engagement can occur.

## Design considerations for stakeholder identification in transdisciplinary research

### Conceptual considerations

Our overall project aim in RECARE is to engage stakeholders for substantive involvement, i.e., to harness knowledge from stakeholders in order to improve outcomes (cf. Fiorino 1990). Such substantive involvement includes co-defining the problems caused by soil threats, identifying management options, and exploring their adoption and implementation. Whether or not this aim is met across the case studies, what we present here is a transferable process to identify stakeholders in order that they can be engaged with. Sustainable solutions to soil threats can only come from a process in which perceptions, experiences, aspirations, stakes and expectations are shared between stakeholders, such that promising ideas emerge for subsequent testing and joint appraisal. This is because ‘solutions’ often imply (co-produced) technologies as well as (co-agreed) organisational, institutional and governance approaches in which technologies are embedded. In short, the rationale for why we need stakeholder engagement in RECARE is simply that researchers cannot develop solutions to soil threats that ‘fit’ without a clear mandate from stakeholders to assist them in countering the threat.

However, a core requirement for engaging with stakeholders in this way is that stakeholders are identified prior to implementing the substantive research stages that seek to generate such knowledge. Where the aim of the research is to go beyond understanding stakeholders to also formulating solutions, it is desirable for the researchers and participants to have as good a starting population of stakeholders as possible before this first data-generation exercise; a baseline understanding of stakeholders (who, and their roles) is needed. In this way, participation does not become onerous in terms of time commitment, and decisions are not shaped before important stakeholders are included. Indeed, stakeholders most appreciate transdisciplinary research processes when they have been involved in formulating issues from the very beginning (Bracken et al. 2014). Similarly, opportunities to enhance the impact of the scientific outcomes are larger when stakeholders are engaged from early in the project, and through the project lifetime (Phillipson et al. 2012).

Identification approaches need to assist researchers in opening up their pre-existing networks, so that there is opportunity to extend participation opportunities to all actors who could be considered to hold a stake in the research problem. Whenever stakeholders are engaged in research, an initial challenge lies in the question of who to engage with, where to draw the boundary between relevant and not relevant, and therefore in judging who should be listened to (Vos 2003). Researchers must consider stakeholders that belong to a range of networks, and not just those that all already know each other (Prell et al. 2009). Focussing only on those previously known and active stakeholders increases the chance of missing hidden, remote or less obvious stakeholders (Reed 2008). Thus stakeholder identification should seek to cover a diversity of stakeholders around a problem in order to allow range of opinions, priorities and options to emerge and be discussed (Prell et al. 2009). Such diversity in perspectives is not necessarily synonymous with diversity in sectors or areas of interest (Cuppen et al. 2010).

Identifying a range of stakeholders necessitates considering all types of stake in a problem or research area. Stakeholders may include actors related to a range of fields of activity that either impact, or are impacted by, the research problem; in the case of soil threats these may include water management, forestry, agricultural production, tourism, etc. Understanding interactions with the problem includes considering the role the stakeholder plays in problem creation, how they experience and are affected by the problem, and what capacity they have to enact solutions. Stakeholders may have different backgrounds, views and perceptions, and may not even agree that there is a problem at all. The primary aim of the stakeholder will influence how they interact with each other and the particular soil threat; for example it matters whether they seek agricultural production or biodiversity conservation (Fisher et al. 2009). Their role and capacity to act may also be influenced by characteristics such as sector, location and ownership, and also willingness to act (Prell et al. 2010; Nieto-Romero et al. 2016).

Stakeholder identification should also be appropriate to sensitivities and dynamics between stakeholders. For example, knowledge exchange and transdisciplinarity is considered more effective when researchers are considered stakeholders themselves, rather than as outsiders or holders of certain powers (Mitton et al. 2007). It is therefore important that the identification stage creates a situation whereby the stakeholders and researchers perceive that they have equal power and authority. In addition, attention must be paid to time constraints of the stakeholder participants, and the potential effects of cultural constraints (Jakobsen et al. 2004). Cultural constraints exist between researchers and stakeholders from different disciplines and backgrounds, such that they have different expectations of theoretical and practical contributions (Fry 2001). Cultural differences also occur between stakeholders from different socio-political backgrounds. For example, acceptance of participation in environmental decision-making is currently poorly institutionalised in Romania (Stringer et al. 2009) and other Eastern-bloc countries due to low social capital and the legacies of communist regimes (Letki 2004).

### Practical design considerations

The RECARE project needed to identify stakeholders in 17 different case study sites, with each case study concerned with a different location and/or soil threat (Table 1). Our stakeholder identification process needed to highlight stakeholders that were affected by, or could affect, the soil threat in the particular location of the case study site. However, in the RECARE project, it was not possible for a single research team-member to identify stakeholders in every case because of the volume of case studies and the range of working languages. Instead, each site is being led by a different case study leader, in a different research institution. Some case study leaders had previously engaged with stakeholders in their case location, and others had not, and all are primarily engaged with physical science disciplines.

A key challenge to identifying stakeholders was in creating a process that was useable and meaningful for all case study leaders. Where case study leaders had previous contact with stakeholders, the identification process needed to encourage them to extend their contacts outside of existing networks. Where leaders had no prior contact, the process needed to stimulate identification of a completely new set of stakeholders. Furthermore, case study leaders had variable experience engaging with more social-science aspects of sustainability science, and with transdisciplinary research.

## Outcomes: the stakeholder identification methodology and its implementation

### Overall approach

**Fig. 1:**
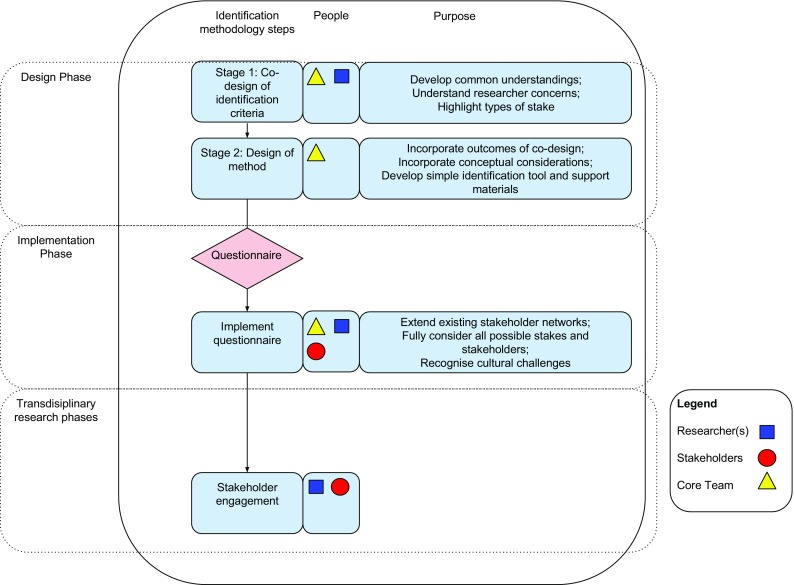
The RECARE methodology for identifying stakeholders for transdisciplinary research, highlighting the phases and steps of the methodology, the people involved, and the purpose of each step. Researchers are shown as *blue squares*; stakeholders are *red circles*; and the core team (paper authors) are shown as *yellow triangles*

Our stakeholder identification methodology is implemented over two phases, both of which take place prior to engaging stakeholders in the transdisciplinary research stages (see Fig. 1). The first phase is a design phase where we worked as a core team (the authors) with the rest of the RECARE researchers in order to develop a tool that they could use to identify stakeholders in the second phase (implementation). Our design phase allowed us to account for conceptual considerations while addressing the practical demands of stakeholder identification for the RECARE project. We thus worked with the case study leaders to ensure their concerns and abilities were accounted for. The resultant tool (a questionnaire) was then implemented by case study leaders with support from a central researcher (the lead author).

### The design phase

The design phase was split into two stages, the first of which was co-design of the identification method with the RECARE case study leaders. As a project team (all RECARE researchers), this allowed us to develop shared understandings of key concepts and ideas, including the purpose of stakeholder identification and engagement. Such space for exploration and negotiation of expectations has been shown to be important in team-managed interdisciplinary research (Jakobsen et al. 2004; Mattor et al. 2014). Having a co-design stage also allowed the core team (authors) to understand the concerns and barriers that the case study leaders perceived around participating in stakeholder identification. As a core team, we could also understand the different types of stake that actors might hold in the various case studies. This understanding meant we could consider how to capture them all when designing the tool. In this way, we could account for the practical challenges presented by the RECARE project.

Co-design centred around a discussion group held between the core team and the case study leaders, followed up by a feedback form (see supplementary material S1). The discussion and subsequent meeting minutes confirmed the challenges of understanding and culture, and of time commitment of case study leaders. A heated discussion was prompted around the issue of the appropriateness of a process to case study locations. Some partners said that they did not feel an identification process was appropriate in the area they worked in, particularly in former Eastern-bloc countries, and therefore expressed reluctance to approach stakeholders. These partners argued that in sensitive case studies, perhaps with turbulent political histories or sensitive soil threats such as industrial pollution, stakeholders would be suspicious of talking to scientists about other stakeholders. Case study leaders were nervous that a structured stakeholder identification process would take too much of their time, and thus cause extra work. Discussion was useful to reassure partners that the time commitment would not be onerous. However, time commitments of case study leaders became an important consideration in designing the protocol. Collectively, these concerns prompted broad discussion on the value of the transdisciplinary research approach, and therefore contributed to a space of developing mutual understanding amongst all case study leaders. In particular, they highlighted the need to develop a protocol that all participants felt comfortable using, and that could not be interpreted as asking stakeholders to report on others.

We took these concerns into the next stage of the design phase, the method design. As a core team, we recognised that case study leaders needed a simple and easy to implement tool, with plenty of support to implement it. The identification tool was therefore designed as a structured, two-part questionnaire (described here, and provided in S2). The questionnaire was chosen as a structured method, that could be accompanied with clear instructions for those case study leaders who felt less confident in implementing the identification process. Part 1 of the questionnaire was designed to characterise a stakeholder, using characteristics highlighted as important by case study leaders during the co-design phase. Part 2 prompted identification of further stakeholders. The co-design stage also highlighted a number of points at which the resulting stakeholder analysis would feed into other parts of the overall project. We therefore designed the questionnaire to provide maximum beneficial information for these other parts of the project. This helped to save time for the case study leaders as it meant that they did not have to repeat work throughout the project.

Support tools were developed alongside the questionnaire in order to address the practical concerns of the case study leaders. Most participants indicated that they would like more information on the purpose of the stakeholder identification in this project. The request was addressed in the support and introductory materials provided to case study partners. These included an instruction sheet that gave an overview of the process, alongside step-by-step instructions (see S3). The instructions were also explained in a PowerPoint presentation with voice recording that was available for partners to download. Two examples of completed questionnaires were provided, and a list of frequently asked questions (FAQs, see S4) was also supplied. The FAQs were intended to answer a range of questions that followed the initial consultation and feedback, and to pre-empt a number of foreseen issues. A participant information sheet (S5) was also provided in order to assist partners in introducing the process to any stakeholders that they contacted. All these documents were made available to partners online, and via email, and partners were encouraged to contact the lead author on email or Skype with any questions or problems.

### The questionnaire

The questionnaire sought to build on and extend the case study leaders’ existing networks by prompting a snowball sampling approach. Case study leaders were asked to fill out the questionnaire as the first stakeholder themselves. Part 1 asked them to provide basic information to characterise themselves (see Table 2). In part 2, they were asked to list the other stakeholders that they already knew. They also provided basic information on their classification, according to their field of activity, role and sector. This section was used to create a snowball sample. Case study leaders were asked to complete a ‘part 1’ form for each of these identified stakeholders in order to characterise them. They were then told to create a sample of at least six of those identified stakeholders. They were instructed to ensure that the six covered the range of roles, sectors and fields of activity that were included in their Part 2 table. The sample formed the core of the snowball sample and were, therefore, approached for a short interview, either in person or on the telephone. In this interview the stakeholder was asked to list further stakeholders that they knew, and thus complete a part 2 of the questionnaire. The case study partner was asked to complete a part 1 for each newly identified stakeholder. Case study partners were asked to continue the snowball sampling process until they started receiving a high number of repeats of the same stakeholders, and no new stakeholders.

**Table 2:** The purpose of the constituent parts of the questionnaire

Questionnaire section	Purpose
Part 1: characterising the stakeholder	
1A	Basic information on the stakeholder, including their size and location
1B	Checks if stakeholder is actually multiple stakeholder, for example if there are local and national branches with different functions
1C	Considers the spatial location and scale of the stakeholders’ interest
1D	Defines the stakeholders interest, including their field of activity, form of role and their sector
Part 2: snowball sample	
2A	Collects information on the other stakeholders that are known to the responding stakeholder
2B	Collects information on stakeholder engagement opportunities that the stakeholder knows of
2C	Collects information on the relevant policies that the stakeholder is aware of

The characterisation section (part 1) of the questionnaire enabled the case study leaders to consider the full range of possible stakes, and uncover potentially hidden stakeholders using the characterisation section (part 1) to select the snowball sample. The instructions provided guidance on ensuring that this sample covered as much diversity as possible in terms of stakeholder characteristics by looking across the table of characteristics. In this way, categorisation of stakeholders accompanied identification in order to ascertain whether or not some types of stakeholder are under-represented or absent (Vos and Achterkamp 2006). Therefore, because we set out to avoid only identifying a small group of similar, connected stakeholders and broaden the stakeholder environment, preliminary categorisation of stakeholders as they were identified served as a control, or iteration, on the identification process. For the same reason, partners were encouraged to include in their sample some stakeholders with which they had not had previous contact. This had the added benefit of raising awareness of the project more broadly.

Further extension of existing networks, and of the range of stakeholders uncovered, was ensured by the lead author. Completed forms were collected by the lead author in order to summarise stakeholders, check for problems or gaps, and provide feedback to the case study partners. This step reduced the time commitment for case study partners as it meant they did not have to integrate or analyse data collected. It also gave an opportunity for some impartial input and iteration. The submission was checked to see if instructions had been followed, at least six identified stakeholders had been contacted, and that part 1 forms had been completed for all stakeholders identified by the partners and their contacted stakeholders. Where there were gaps in the data, the partner was contacted and asked to submit the missing data. When submissions were considered complete, the lead author input all data into an Excel spreadsheet. The tables were then considered by the lead author, in combination with her understanding of the soil threat. Administrative levels, topics or sectors that appeared to be underrepresented were identified. Suggestions were then made to case study partners via a written report about gaps they may wish to fill, and what function this would play in their stakeholder engagement. In future, allowing time for face-to-face feedback and discussion would be an improvement.

Issues of sensitive dynamics between stakeholders were built into the questionnaire’s design. We ensured all questions could be completed by the case study partner using publically available information, or by short, non-intrusive questions to the stakeholder in question. By only requesting publically available information, we hoped to ensure that no stakeholder felt that the researcher was asking sensitive questions. It also meant that the case study partner could still complete the questionnaire in the case of feeling too uncomfortable to contact the stakeholder. Furthermore, all case study leaders were asked to conduct the identification prior to the first session where they would be trained in the transdisciplinary workshop approach. This meant that stakeholders would be identified in plenty of time to be engaged in the research, and not once this transdisciplinary phase was already under way. Finally, where case study leaders did choose to contact stakeholders during identification, the process offered an opportunity to introduce the project and the research team.

### Implementation of the questionnaire

The questionnaire was generally well implemented by case study partners, and extended the number and diversity of stakeholders known in most cases. Of the 17 partners who submitted, 10 had accurately followed the instructions and provided between 10 and 49 part 1 completed questionnaires. The remaining partners submitted some questionnaires, but had not followed the instructions in full. In some cases, this meant that no further details had been submitted for stakeholders identified in the first snowball. These partners were contacted and the additional details were requested. Table 3 demonstrates that in at least 9 of the cases, the snowball sample approach extended the number of stakeholders known (Table 3, section A). Furthermore, the final set of identified stakeholders covered a wide range of fields of interest in addition to the farmers and land owners that partners initially predicted, including education, community development, insurance, and traffic management (Table 3, section B). Identified stakeholders also represented a range of administrative levels (Table 3, section C).

**Table 3:** Stakeholders identified by the questionnaire process

Case study	A. Stakeholder numbers		B. Field of activity										
	Initial^a^	Final^b^	Education	Forestry	Env. protection/conservation	Agriculture	Recreation	Research and development	Product/commodity exploitation	Water management	Land use policy and planning	Community development	Other
1	33	44	Y, X		Y, X	Y, X	Y, X	Y, X	Y, X	Y, X	Y, X	Y, X	Insurance
2	^d^	19		X	X			X	X		X	X	Fire management
3	^c^	8			Y, X	Y, X		X		Y, X	X	Y, X	Cultural Heritage
4	4	10	X		Y, X				Y, X			X	Tourism water end users construction
5	26	46			Y, X	Y, X		Y, X		X	Y, X		Traffic management
6	^c^	9			X	X		X			X		
7	^c^	6				X	X	X		X			
8	^c^	48		X	X	X	X		X		X		Soil
9	^d^	35			X	X	X		X	X			Insurance companies Road authorities
10	9	13			Y, X	Y, X				X	X	Y, X	Land owners
11	^d^	31				X				X	X		
12	0	6		X	X	X		X					
13	^d^	49	X		X	X		X	X	X	X	X	
14	6	26	X		X	Y, X		Y, X		Y, X	Y, X		
15	^d^	6	X	X	X	X	X	X	X	X	X	X	
16	6	16			X	Y, X		Y, X			Y, X	X	
17	^c^	19			X	X	X	X		X	X		Military Landscape protection designation

Case study	C. Level																
	Local	Sub-National	National														
1	X	X	X														
2	X	X															
3	X		X														
4	X	X															
5	X	X	X														
6	X	X	X														
7	X	X															
8	X	X															
9	X	X	X														
10	X	X															
11	X	X															
12	X	X	X														
13	^c^	^c^	^c^														
14		X	X														
15	X	X															
16	X	X															
17	X	X	X														

*Y* field of activity included in initial list of stakeholders from the case study leader at the beginning of the process (information can only be provided where superscript letters d and c are not used in the ‘stakeholder numbers’ columns). *X* field of activity and level included by the end of the process

^a^Number identified by case study partners on their initial completion of Part 2 of the questionnaire

^b^Number of stakeholders identified at the end of the process

^c^Information not provided by case study partner

^d^Information was provided directly in Excel format, rather than in questionnaire form, meaning parts of the process were not visible to the core team

The process was most effective when it was implemented by engaged and proactive case study leaders. The lead author was contacted multiple times by a number of researchers with questions about the process. While these case study partners initially seemed more uncertain, they engaged with the process and were proactive in seeking help. These researchers submitted completed questionnaires on time, and identified a large number of stakeholders from diverse categories (e.g., case studies 2, 5, 9 in Table 3). Therefore, engaged and proactive case study partners seemed better at using the questionnaire to produce useable outcomes. Where partners submitted on time, and/or in communication with the task leader, the task leader was able to control the quality of data submitted, fill in gaps and provide feedback to case study partners as the process evolved. Such iteration and feedback was more limited in partners that submitted late. However, in all cases, a written feedback report provided a further round of input and feedback.

The analysis was used in the training for case study partners on stakeholder interaction organised in Wageningen, The Netherlands, in September 2014. In the training sessions, case study partners were encouraged to think about which stakeholders they might engage with, and how. Each case study partner was given a summary of 7–15 case study stakeholders from their own list with information on size, topic, role, sector, and aim included. The case study partners were asked to estimate the stakeholders’ motivation for and influence on the sustainable management of the local soil threat. This was done by placing each stakeholder onto an influence-motivation matrix (see for example Schwilch et al. 2009). The exercise further helped to identify if any important stakeholder groups that should be involved were missing, and may need to be approached specifically. As the identification information is being used in the ongoing RECARE project, case study partners are making suggestions for future changes to the identification protocol. The column “aim” contained the most useful information for this exercise, and facilitated the placement of stakeholders onto the matrix. However, as a recommendation from the training, the case study partners suggested to add to the protocol a specific question on the “type of stake in the soil threat”. Such a question would have an open response, and is therefore more descriptive, allowing a large range of responses, including, for example: “grows crops in the soil”; or “has to remove soil sediment from drainage ditches when they are blocked”. In this way, the case study leader would be encouraged to consider the explicit link between the stakeholder and the research question, thus assisting their understanding (and identification) of stakeholders.

Some feedback on the stakeholder identification process was obtained from case study partners during the second project plenary meeting held in Padova in March 2015. Case study partners acknowledged the clear and formalised process of identifying stakeholders. Case study partners reported that in least 12 out of the 17 case study sites the stakeholder analysis enabled to identify new stakeholders, beyond those known by the case study partner before (see Table 3, section A). Furthermore, the funders’ evaluation of the project (September 2015) was complimentary about the stakeholder identification report. It highlighted that the individual recommendations made to case study partners were very relevant.

## A transferable stakeholder identification approach for transdisciplinary research

The methodology outlined in this article enabled the identification of a diverse range of stakeholders for engagement at the appropriate (early) time (Stauffacher et al. 2008) in the transdisciplinary research project. We have created a process that is specifically designed to identify stakeholders so that they are known and visible for consideration for inclusion in the transdisciplinary stages of the research process. Rather than using an initial stakeholder workshop or event to identify further stakeholders, we guide researchers through a structured approach for early identification. In this way, stakeholder identification is designed into the research project so that it maximises the stakeholders’ opportunities to contribute and shape the research (as per Fiorino’s instrumental reason) (see Fig. 1). For those scientists with pre-existing stakeholder networks, it helps to diversify and broaden these, ensuring that scientists are able to consider participants that represent a range of knowledge and experience (as per Fiorino’s normative and substantive reasons). For those without networks, or with more uncertainty around conducting such ‘social’ research, the protocol provides clear and transparent steps, with support on approaching stakeholders to identify a population from which to consider participants. Importantly, the questionnaire is non-intrusive for stakeholders, catering to cases with cultural constraints or sensitivities around approaching stakeholders. Indeed, the process can create an opportunity to introduce the research to stakeholders.

While our questionnaire seeks to increase and diversify the stakeholders known to the research team, there remains potential for bias to be introduced by the implementing researcher. We don’t know the extent to which their individual values or existing networks have influenced the identification process, and therefore influences the perspectives (Lang et al. 2012). This issue of bias is in part moderated by the core team; in cases where the questionnaire yielded limited stakeholder diversity, recommendations from the central researcher served to prompt further reflection by the case study leader. However, subjective identification of researchers can influence the outcomes of the research project (Fletcher 2007). Follow up research, potentially as part of the on going project, should seek to evaluation how differences in implementation have influenced identification, and therefore engagement and participation, and therefore have influenced the overall research project (Stokols et al. 2008).

Bias should be addressed by seeing our questionnaire as a foundational stage in the overall transdisciplinary project. The questionnaire cannot ensure that a diverse range of stakeholders and perspectives are included in the transdisciplinary research stages, nor that the dynamics between stakeholders are managed; it merely provides a population of stakeholders from which to sample, and an understanding of what dynamics may exist. In case studies with a low population of stakeholders, partners could invite all of them to participate in the stakeholder platforms. In cases with a high number of stakeholders, a further sampling stage may be necessary. In our project, this is being handled at the individual case study level, with guidance from specialised researchers. However, a sampling stage could be considered as a future add-on to the identification methodology. It should draw on approaches that seek to sample based on diversity in stakeholder perspectives (e.g. Cuppen 2012). By actively seeking to invite stakeholders with diverse perspectives, there is a higher likelihood of moving away from the researcher’s perspectives, and thus a higher chance of removing their identification bias, though the sampler would need to be sensitive to problematic relationships (and their management).

As part of its role as a foundational transdisciplinary research stage, the questionnaire and its design process acts as a site of integration and the negotiation of common understandings between project members (see Jakobsen et al. 2004). In terms of the questionnaire, clear instructions and support materials meant that even under-confident scientists with no prior engagement with social sciences were able to conduct comprehensive identifications. This is a benefit of our approach as it has helped to build interdisciplinarity within the researchers in the project, in that physical science specialists could engage in social science processes. In addition, the process of designing the questionnaire provided a forum for all researchers (from all disciplines) to understand each other. Such mutual understandings included the idea of what a stakeholder is, and why they should be engaged. But also extended to practical challenges, including cultural barriers to approaching stakeholders, and the problem of researcher time limitations. As a core team, we were able to respond to concerns in the design of the questionnaire. But we were also able to be transparent about the time commitment involved in the set-up stages of the project, and the overall benefit of this to outcomes of the project. We believe this process increased the engagement of the researchers in the process, and led to a well-implemented questionnaire.

From our experience, we would argue that a key tool to facilitate mutual understanding in the project team is the use of support and advice mechanisms. These support tools allowed the researchers to interact easily with the process. In our project, due to the large number of cases and geographical distribution, email and telephone were the most appropriate solutions. However, researchers could consider whether small group meetings or closer collaboration with case study partners would also be possible. Such decisions should be made in collaboration with the case study partners, taking into account their requirements, concerns and availability. Thus, the coordinating role, for facilitating refinement of options, and providing support is important. This coordinating person does not need to be a social scientist. However, it is important that this person is confident in stakeholder engagement because of their positioning as an ‘expert’ for the task

The exact design of the questionnaire and the way stakeholders are characterised may be of limited transferability; rather it would need to be tailored to the context of a specific project and problem. The ways in which stakeholders are characterised (location, topic, sector, etc.) may need to be reviewed for the elements that are important for the specific research project. Additionally, the answer options for these multiple choice questions should be specifically designed for the case study or studies included. For example, asking if someone is a forest product certifier may be deemed less relevant in case studies without influence from the forestry sector. In cases of doubt, we recommend that more options are included. In larger projects, such refinement would be the task of a core team.

Indeed the transferable methodology is the two-stage process for refining and implementing the questionnaire, and its positioning within the overall project (Fig. 1). Case study partners should be engaged early in the project so that mutual understanding can be achieved. We found that a face-to-face discussion format was extremely beneficial in fostering common understanding. Follow up with a feedback form highlighted remaining areas of concern and misunderstanding that could then be addressed in the support materials. Such engagement should also provide an opportunity to consider how support will be provided, and to refine parts of the protocol that refer to the characterisation of stakeholders. The feedback form was most helpful for this. The core team should then refine the protocol, provide detailed instructions and maintain ongoing communication with the case study partners throughout the process. The same person or team should integrate results, provide feedback and demonstrate the applicability of the results.

## Conclusions

The methodology outlined in this paper provides a useable, systematic approach for identifying stakeholders at the start of transdisciplinary projects. It is designed to facilitate identification of a broad range of stakeholders around a given problem and/or location. The methodology comprises two phases: design and implementation of a questionnaire. We applied it to a large transdisciplinary research project, so that multiple researchers can identify stakeholders in a broad range of problem contexts. The questionnaire allowed information to be collected in a systematic way across all case study sites, such that members of a core team could gain a cross-case understanding. The co-design stage of our process meant that the questionnaire was designed to account for time constraints of researchers, and to be sensitive to a range of social contexts and cultural constraints. It also served as an integration process for developing common understanding of the research process throughout the project team. The support materials provided made the questionnaire easy to use for researchers with very little prior experience of social research, provided they were committed to the process. The questionnaire and its design/refinement process can be a useful and transferable tool for increasing transparency in the early stages of transdisciplinary research. In particular, researchers can be more explicit about how stakeholders were identified, and in how they then drew on the stakeholder population for inclusion in a transdisciplinary project. Therefore, we can better understand who has had opportunity to shape research outcomes.

We recommend that our questionnaire can be refined for use (during the design phase) whenever there is a need to identify stakeholders prior to considering who to include, how, and before beginning to shape research or projects. The problem context will shape how stakeholders may need to be characterised. This should be adapted during an initial design phase so that contexts and researcher concerns can be accounted for in the refined questionnaire. The adapted questionnaire can be used in single case study sites, or in multiple cases (such as in our example). In multiple-case research, it is useful to have a core team to coordinate all stages of the process, and particularly to provide support and iteration of results.

## Electronic supplementary material

Below is the link to the electronic supplementary material.

Supplementary material 1 (DOCX 71 kb)

Supplementary material 2 (DOCX 34 kb)

Supplementary material 3 (DOCX 136 kb)

Supplementary material 4 (DOCX 153 kb)

Supplementary material 5 (DOCX 134 kb)
