# Supplementary material for: An applied methodology for stakeholder identification in transdisciplinary research
Source: Sustain Sci. 2016 Jul 26;11(5):763–75. doi: 10.1007/s11625-016-0385-1 (PMC6106094; doi:10.1007/s11625-016-0385-1)
Supplement: Supplementary file 1 — Supplementary material 1 (DOCX 71 kb) [file 11625_2016_385_MOESM1_ESM.docx]

Name:

Email address:

Partner:

Case Study (if applicable):

**Please feel free to add additional comments or thoughts that are not covered in this questionnaire!**

## Understanding

A stakeholder is anyone (individual or organization) that is impacted by, or can affect, a decision or action. They could work at a local (farm) level, or may work on broader scales (e.g. regional, national, etc.).

**I understand the term stakeholder well enough:**  agree/disagree

**I would like more information to understand this term and its relevance to my case study:** agree/disagree

**Comments** (e.g. what kind of questions do you have?):

A stakeholder analysis will identify stakeholders, understand their role and influence in soil management decisions and actions. It will therefore identify actors for ongoing knowledge exchange and involvement in the RECARE project.

**I understand the aim and purpose of a stakeholder analysis for my case study and the broader RECARE project:** agree/disagree

**I would like more information to understand the aim and purpose of a stakeholder analysis for my case study and the broader RECARE project:** agree/disagree

**Comments** (e.g. what kind of questions do you have or how can this analysis be more useful to your case study?):

## Input to the Stakeholder Analysis

We are thinking about who plays a role in determining whether or not a soil threat management system will be adopted and what their role is.

**Which of the following stakeholder roles are important in your case study site?**

| **Form of influence** | **Highly important** | **Slightly important** | **Not important** | **Unsure** |
| --- | --- | --- | --- | --- |
| Providing information |  |  |  |  |
| Providing education and/or training |  |  |  |  |
| Providing finance |  |  |  |  |
| Providing tools and equipment |  |  |  |  |
| Creating market opportunities |  |  |  |  |
| Creating or enforcing legal requirements |  |  |  |  |
| Popular pressure/campaigning |  |  |  |  |
| Implementing land management |  |  |  |  |
| Others (please specify) |  |  |  |  |
|  |  |  |  |  |
|  |  |  |  |  |
|  |  |  |  |  |

We are also interested in understanding the extent to which stakeholders are able to influence actual land management.

**Which of the following characteristics determine the effectiveness of stakeholders in influencing decisions and actions in your case study area?**

| **Characteristics of effectiveness** | **Highly important** | **Slightly important** | **Not important** | **Unsure** |
| --- | --- | --- | --- | --- |
| Stakeholder’s location |  |  |  |  |
| Their sector (e.g. NGO, private, etc.) |  |  |  |  |
| Their role (land manager, consumer, etc.) |  |  |  |  |
| Their interest (food production, conservation) |  |  |  |  |
| Form of role (as above) |  |  |  |  |
| Scale of their work (e.g. local, district, national, etc.) |  |  |  |  |
| Financial assets |  |  |  |  |
| Size of organization |  |  |  |  |
| Connections with other key stakeholders |  |  |  |  |
| Frequency of contact with others |  |  |  |  |
| Quality of contact with others |  |  |  |  |
| The farmer’s opinion or relationship with the stakeholder |  |  |  |  |
| Other suggestions (please specify) |  |  |  |  |
|  |  |  |  |  |
|  |  |  |  |  |
|  |  |  |  |  |

## Practicalities

**What is the approximate size of your case study area? (e.g. km^2^)**

**How many individual farmers (or land managers) are within your study area?**

**Would you be able to spend approximately 1 hour with a sample of stakeholders to complete the stakeholder survey?**

**If not, please provide some information on the barriers or your reluctance:**

**Can you do it before the end of March (if you are given the forms before the end of February).**

**If not, when would you be able to do so?**

Following this round of data collection, it might be desirable to contact some farmers to ask some more detailed follow-up questions. This could be done via telephone or face to face. **Would you be willing to conduct such questioning if necessary?**

**If not, please provide some information on the barriers or on your reluctance:**

**Would you prefer to fill in directly to Excel, or have a paper questionnaire?** Paper/Excel
