# Supplementary material for: An applied methodology for stakeholder identification in transdisciplinary research
Source: Sustain Sci. 2016 Jul 26;11(5):763–75. doi: 10.1007/s11625-016-0385-1 (PMC6106094; doi:10.1007/s11625-016-0385-1)
Supplement: Supplementary file 2 — Supplementary material 2 (DOCX 34 kb) [file 11625_2016_385_MOESM2_ESM.docx]

**Form number:** Please number every form (e.g. 1, 2, 3).

**Date:**

**Form completed by (select one):** Researcher/stakeholder/researcher and stakeholder

# Part 1: Stakeholder Characterisation

**A stakeholder is anyone who can affect, or be affected by, an action or a decision. They may have different interests and act at different scales and some may be hidden. They might be an individual, a member of an organization, or an organization itself.**

*This section is to be completed for each stakeholder identified. It does not have to be done by the stakeholder themselves, but the researcher can complete, looking up missing information or contacting a stakeholder to ask questions for any missing information.*

## 1A. Basic information

1A.1 Name of stakeholder: *e.g. Bob Smith*

1A.2 Name of stakeholder’s organisation: *e.g. University of Here. Sometimes the stakeholder name will be the same as the respondent’s name.*

1A.3 Contact details: *Phone, email or postal address depending on the best/easiest way to contact them.*

1A.4 Physical location: *Village or town – full street address is best, but not necessary if the respondent does not wish to provide it.*

1A.5 Website (where applicable):

1A.6 How big is the stakeholder?

Individual ☐

2-10 people ☐

11 – 50 people ☐

50+ people ☐

## 1B. Organisational structure

1B.1 Is this a sub-office of a larger organization? *E.g. is this the regional Environment Agency (which also exists as a national body)? If so, then it is a sub-office of a larger organization.*

Yes ☐(continue to 1B.2)

No ☐(continue to question 1B.4)

1B.2 If yes, could the larger organization also be considered a stakeholder? *Are they also interested in some way in what happens on the site, do they influence it, or are they also affected somehow?*

Yes ☐(continue to 1B.3)

No ☐(continue to question 1B.4)

1B.3 If yes, please also complete a form for them. Form number:

1B.4 Does this stakeholder have any sub-offices? *E.g. does the regional Environment Agency also have district offices beneath it?*

Yes ☐(continue to 1B.5)

No ☐(continue to section 1C

1B.5 If yes, could they also be considered a stakeholder? *E.g. perhaps one of the district offices covers the case study area?*

Yes ☐(continue to 1B.3)

No ☐(continue to section 1B.4)

1B.6 If yes, please also complete a form for them. Form number:

## 1C. Area of focus

1C.1 Is this stakeholder’s physical area interest defined by ecological or administrative boundaries? *An administrative boundary might be a district, a region, a state, a canton, or even a field. An ecological boundary might be a forest or a river basis. The administrative and ecological boundaries may not coincide, but in some cases they may (in which case chose both). For example, a river basin is often an ecological unit and an administrative unit (where there is a river basin authority).*

Ecological ☐ (continue to 1C.2)

Administrative ☐ (continue to 1C.3)

Both ☐ (complete 1C.2 and 1C.3)

1C.2 If ecological, please describe the physical area that they are interested in. *E.g. The Here Heath.*

1C.3 If administrative, please specify the physical area that they are interested in. *E.g. Here district.*

## 1D. Defining the Stakeholder’s Interest

1D.1 What is the stakeholder’s topic of involvement in the area? Please tick all that apply, and then indicate their main topic by underlining this topic as well.

Education ☐

Forestry ☐

Environmental protection and conservation ☐

Agriculture ☐

Recreation ☐

Research and Development ☐

Product/commodity exploitation ☐

Water management ☐

Land use policy and planning ☐

Community development ☐

Other☐ Specify:

1D.2 What is the stakeholder’s role or form of interest? Please tick all that apply, and then underline the primary or most important role.

Land owner ☐

Land manager ☐

Employee on a farm or in a forestry operation ☐ (list example tasks):

Consumer of produce ☐

Consumer of other services (recreation, water, etc.) ☐

Provider of information to the public ☐

Provider of information / management advice to farmers or forest owners☐

Regulation and enforcement ☐

Equipment and/or tool provision ☐

Creating market opportunities for products ☐

Retailer of products ☐

Providing finance to land managers/owners/workers ☐

Assistance in gaining access to public funding for land management ☐

Campaigning ☐

Community leaders ☐

Safety and/or Security provider ☐

Provider of other services ☐

Constructor (infrastructure and/or buildings) ☐

Product certification (e.g. organic, FSC) ☐

Other ☐ Specify

1D.3 What sector does this stakeholder belong to?

Government ☐

NGO ☐

Private Sector: retail ☐

Private Sector: industry ☐

Private Sector: other ☐

Academic ☐

Private individual ☐

Public enterprise ☐

Civil Society ☐

Other ☐ Specify

1D.4 What is the main aim of this stakeholder regarding land management in the study site? (free text) *E.g. This person/institution provides advice to farmers on: production/yield implements, water management, soil managements, products, etc.*

# Part 2: Snowball Sample

*This section should be completed with a sample of stakeholders in order to extend the analysis beyond what the researcher already knows. For every identified stakeholder, a separate form (part 1) should be completed – hopefully facilitating a bit of contact between researcher and the new stakeholder.*

## 2A. Other Stakeholders

2A.1 Please list with the stakeholder the other stakeholders that they identify. These may be people or organisations that they work with, have contact with, or that they feel influence (or are influenced by) their own work in some way. Continue on a separate sheet if necessary (see end).

*The researcher should help the stakeholder in two ways. Firstly, using the prompt sheet to identify the right word to put in each cell. Secondly, use the categories to prompt the stakeholder. For example “I see you have not mentioned anyone dealing with water management. Do you know of any water management people or organisations that might be interested in or affected by your land management?”*

| Stakeholder | Topic | Role | Sector | Form number |
| --- | --- | --- | --- | --- |
|  |  |  |  |  |
|  |  |  |  |  |
|  |  |  |  |  |
|  |  |  |  |  |
|  |  |  |  |  |
|  |  |  |  |  |

## 2B. Current Stakeholder Engagement

*The stakeholder should answer these questions only for themselves, and not for the stakeholders that they identified in section 2A.*

2B.1 Which information channels do you usually use, or which events / meetings do you visit if you want to obtain information, discuss or exchange experiences on questions related to land management? Please specify.

Networks (e.g. farmer organisation, regional working group ‘no-tillage’, etc.):

Newsletters (e.g. quarterly newsletter of the Ministry of Agriculture, newsletter of the national association of organic farmers, etc.):

Events (e.g. agricultural fair, workshops of extension service provider, village meeting, etc.):

Online communities (e.g. online discussion fora, websites, social media, etc.) :

Clubs, organisations or societies (e.g. Young Farmers Association, Women’s Institute):

Others (please explain):

## 2C. Current Policy Awareness

*The stakeholder should answer these questions only for themselves, and not for the stakeholders that they identified in section 2A.*

2C1 Please list with the stakeholder, the policies, plans or programmes that the stakeholder is aware of that influence their work. If possible, include the colloquial and official names of the policy, and indicate if they are EU or national, and how they affect the stakeholder’s work. Continue on a separate sheet if necessary (see end)

| Common name | Official name | EU/National | Reason important to stakeholder |
| --- | --- | --- | --- |
|  |  |  |  |
|  |  |  |  |
|  |  |  |  |
|  |  |  |  |
|  |  |  |  |
|  |  |  |  |

## 2A. Other Stakeholders (continuation table)

| Stakeholder | Topic | Role | Sector | Form Number |
| --- | --- | --- | --- | --- |
|  |  |  |  |  |
|  |  |  |  |  |
|  |  |  |  |  |
|  |  |  |  |  |
|  |  |  |  |  |
|  |  |  |  |  |
|  |  |  |  |  |
|  |  |  |  |  |
|  |  |  |  |  |

## 2C. Current Policy Awareness (Continuation Table)

| Common name | Official name | EU/National | Reason important to stakeholder |
| --- | --- | --- | --- |
|  |  |  |  |
|  |  |  |  |
|  |  |  |  |
|  |  |  |  |
|  |  |  |  |
|  |  |  |  |
|  |  |  |  |
|  |  |  |  |
