# Supplementary material for: An applied methodology for stakeholder identification in transdisciplinary research
Source: Sustain Sci. 2016 Jul 26;11(5):763–75. doi: 10.1007/s11625-016-0385-1 (PMC6106094; doi:10.1007/s11625-016-0385-1)
Supplement: Supplementary file 3 — Supplementary material 3 (DOCX 136 kb) [file 11625_2016_385_MOESM3_ESM.docx]

RECARE Stakeholder Identification Instructions

# Overview

We are aiming to build a web of stakeholders within the different soil threat topic areas. We would like to identify as broad of an array of diverse stakeholders from each case study area as possible. The process of identifying stakeholders starts with you, according to the following steps:

Step 1: Fill out the Questionnaire. Your answers to Part 2 will be used to expand the stakeholder web. For each stakeholder you identify, please fill out a new Questionnaire (Part 1 only).

Step 2: From the group of stakeholders you identified, select 6 to contact and formally guide through Part 2 of their Questionnaire.

Step 3: Based on their answers to Part 2, please fill out a new Questionnaire (Part 1 only) for each of the stakeholders they identify.

Part 4: If more stakeholders are needed, repeat Step 2.

The process

Every identified stakeholder should have the Part 1 section of the questionnaire completed for them. This DOES NOT mean that you have to contact every stakeholder – you can probably fill this information in very quickly about them, or look it up as you need to. You might chose to contact a stakeholder if you are not sure, or if you would like to use it as an excuse to introduce yourself and the RECARE project (see Stakeholder Engagement Guidelines). These instructions will help you in completing Part 1.

Part 2 needs to be completed with the stakeholder, but only for a sample of those identified. These instructions will help you to decide which stakeholders, and will help in completing Part 2.

Questionnaires can be completed by hand or on a word document. Those completed by hand should be scanned and emailed to me, or mailed (registered delivery).

Before you send them back to me, please check that I will be able to understand what is written. For most answers, the multiple choice format means that you won’t need to do any translating. If you chose to translate the form before using it, please keep the answers in the same order. For any free text, please provide a short translation.

# Step 1: You (be a stakeholder)

**Use 1Questionnaire.docx.** Consider yourself as a stakeholder and complete the entire form (parts 1 and 2).

1. Complete Part 1 using your details and information.
2. In part 2, list those stakeholders that you already know. Use the prompt sheet to practice how it will work when you are interviewing stakeholders. Ask yourself the questions given on the prompt sheet, and add more stakeholders as you can. **Think about whether you have included less-obvious or marginalized stakeholders.** You may find that you think of stakeholders that you have not previously spoken to or contacted. Some topics may not be relevant to your case study, but that’s OK. Try to think of stakeholders that are relevant.
3. For each stakeholder that you identify, complete a further form Part 1 and write the number in the cell. Please look up information if you aren’t sure; please try not to leave blanks or guess. You could search online or contact a stakeholder to ask for information if you are not sure of something. *In section 1D, do the best you can, and verify with the stakeholder if you contact them in Steps 2 and 3.*

# Step 2: Snowball sample

Look at the list of stakeholders that you have created. Your aim is to make a selection of stakeholders that offers a diverse range of topics, roles, and sectors. I would suggest that you need at least one from each of the topics that are relevant to your case study. You should also try to make sure that you have a range of roles and sectors covered as well.

**We are aiming for you to speak to 6 stakeholders in total to ask them to complete this form.**

1. If you have identified a diverse range of stakeholders in your own form, you may wish to just choose 6 straight away. Make sure your 6 represent as much diversity in topic, role and sector as possible. You may wish to include some that you have not spoken with before in order to introduce yourself and the RECARE project.
2. If you have identified only farm level stakeholders in your own form, you may wish to contact a smaller number of them (representing as much diversity between them as you can).
3. If you are not sure what to do, or would like a second opinion on your sample, contact me.

# Step 3: Contact your sample

Visit or talk on the telephone with your sample in order to *check the details of Part 1*, and to complete Part 2 from their perspectives.

1. Contact the stakeholder to arrange a short meeting. When contacting stakeholders at the farm level, please do not ask solely for the farmer, or for Mr. Name. Particularly if the farm is a family business, it is important to include those members of the family that might be involved in decision making on the farm. If we only speak with the most visible farmer, we may not capture organisations that target young or female farmers. Perhaps ask to speak to the family, or try to include various family members in the sample rather than always the most visible person.
2. Use your contact with stakeholders as a way to introduce the RECARE project. Take with you the **local language project fliers**, and the **participant information sheets (2ParticipantInformationSheet.docx).** The participant information sheets are very important and every person you interview for this stakeholder analysis must have one to keep! You will need to translate it into local language first.
3. Use the participant information to explain that you are currently identifying stakeholders in the project, and would like to ask for their participation in that identification process. Please make sure you cover all of the points verbally with the stakeholder so that they understand what the RECARE project is, what the stakeholder analysis is, and what their participation is.
4. Check the information in Part 1 with the stakeholder (in particular 1D). Then help them to complete part 2. Use the **prompt sheets (3PromptSheet.docx)** available. If the stakeholder asks not to be named, please cross off names on forms before submitting them to me. A black marker pen works well for this.

**Some respondents will list stakeholders that have already been listed by you or other stakeholders. You MUST list these on the stakeholder’s form, even if they are repeats. It is useful to me to see how often a stakeholder is referred to by others!**

# Step 4: Round 2?

Now that you have information from a sample of stakeholders, you need to think if you should do another round of sampling. Firstly, have you done 6 stakeholders?

1. If no, then you will need to do some more!
2. If yes, look at the complete list of stakeholders that you and all your respondents named. Is there starting to be a large amount of overlap between the stakeholders being identified? If so, you probably have enough. If not, please do a further round of sampling.
3. If you need to do more, look at your complete set of identified stakeholders, and start again at Step 2.

**When talking with a newly identified stakeholder, DO NOT name the person who identified them unless you have specific permission to do so.** Naming someone you have already identified would break the promise you have made of confidentiality (see participant information).
