# Supplementary material for: An applied methodology for stakeholder identification in transdisciplinary research
Source: Sustain Sci. 2016 Jul 26;11(5):763–75. doi: 10.1007/s11625-016-0385-1 (PMC6106094; doi:10.1007/s11625-016-0385-1)
Supplement: Supplementary file 4 — Supplementary material 4 (DOCX 153 kb) [file 11625_2016_385_MOESM4_ESM.docx]

# RECARE Stakeholder Analysis: Frequently Asked Questions

## Defining Stakeholders

#### What is a stakeholder?

A stakeholder is anyone who can affect, or be affected by, an action or a decision. They may have different interests and act at different scales and some may be hidden. They might be an individual, or an organization or group.

#### Do stakeholders include anyone who is affected by, or can influence, the soil threat?

In short, yes. We don’t expect you to identify every individual person and organization – in some cases this would make for an unrealistically large analysis. But we do want to encourage you to think beyond the farmers themselves, to understand the different kinds of actors that might play a role, what that role might be, and give some consideration as to whether they should be involved further in RECARE.

#### Why do we need to look beyond just those people who directly work on the land, or those that I am already aware of?

Those who work on the land will be very important stakeholders, and many of you already have good relationships with a range of stakeholders. We are not asking you to forget about these. We do want to encourage you to look beyond these though; land managers usually make their decisions about land management based on a range of criteria (maybe unconsciously) such as what their neighbours are doing, whether there is market demand for a particular product, whether they have access to information and training on a particular technique, etc. Other stakeholders help to shape those criteria, and so we need to identify such stakeholders in order to understand their role and engage them in sustainable land management.

#### What use is the stakeholder analysis to the RECARE project?

The stakeholder analysis will help for the case studies going forwards in completing the workshops under WP4. It should also feed into WP9 (policy analysis). During the workshops in WP4, there is likely to be more analysis of the how much influence or power each stakeholder has.

#### What overarching analysis will be done with the stakeholder analysis?

Most of the analysis done in the stakeholder analysis will be useful to case study partners and will feed directly back into their work. I will also summarise the process and the results in deliverable 4.1, which is due in July. To take it further, it will be interesting to see which types of stakeholders are most engaged in RECARE as the project progresses. I would also like to compare between soil threats and locations, tying into characterisations of what constitutes ‘farming’ in each location, in order to see what differences there are in terms of who is a stakeholder, and why.

## Sampling and approaching stakeholders

#### How do I know which stakeholders to approach?

Use the instructions that have been sent. These instructions will help you think about which stakeholders you need to know more about, and how many of what category to contact. It also explains to make sure you do not automatically include only the most visible stakeholders and respondents.

#### I contacted a stakeholder to talk to them, and they asked me lots of questions about the stakeholder analysis and the RECARE project. What do I do?

Hopefully, the participant information sheet and your own knowledge (and these FAQ’s) will be sufficient to answer most questions. Please do use these materials to their full extent. If questions still remain, please do feel free to offer to send their questions to me, and I will answer them as fully as possible.

#### I contacted a stakeholder to talk to them, and they are reluctant or have refused. What do I do?

Sometimes respondents are reluctant to engage, or may even refuse. Reasons may include a lack of time, or even lack of interest in the project. Try to ascertain why the respondent is reluctant and see if you can persuade them; use the information sheets and dissemination leaflets to answer their questions. If the participant refuses, this is OK – don’t hassle them about it. You can try another stakeholder – maybe even someone else within that farm or organization. Try to make sure that your replacement stakeholder keeps the gender balance. If a participant refuses, be sure to still include them in future communications in case they wish to be involved later on.

## Conducting the analysis

#### I can fill out much of this information without having to contact the stakeholders. So why am I being instructed to sample and contact stakeholders?

The contact with stakeholders serves many functions. From the point of view of the stakeholder analysis, it allows us to complete the information about them that we didn’t already know, and (importantly), allows us to ask stakeholders for further stakeholders. By asking a stakeholder who they interact with, and on what basis, we can conduct a snowball sample and uncover stakeholders that we might not have been aware of. In addition, contacting stakeholders allows you to build a relationship with them (if you don’t already), inform them of the project, and invite them to workshops.

#### Do I have to contact all stakeholders?

No, you do not have to. You are only contacting a sample in order to ensure that you have identified a fairly comprehensive collection of relevant stakeholders. You will not need to ask questions of all of them, and therefore do not need to contact them all to conduct the analysis. However, you may wish to do so to introduce yourself and the project, and to invite stakeholders to workshops.

#### Not all of topics/roles/sectors are relevant to my case study – what should I do?

The options included here are to make you think about whether or not there are relevant actors that have not previously been included. However, use them as a prompt, rather than thinking that you must find an actor for each category. Only include actors that really have a stake in the case study area. Bear in mind that the forms were written to cover options for 17 different case studies. So some options may not be relevant to your case.

#### Do I have to answer every question for every identified stakeholder?

No – you should follow the question guide in order to determine which question you need to answer for each stakeholder. Please stick to the question guide – it is important to be consistent in the information that we are collecting within and between case study sites.

#### Can I ask stakeholders questions that aren’t included in the analysis?

Yes, of course. If you think that they are relevant to determining who the key stakeholders are, or shaping what makes a key stakeholder, please do feel free to include this information in your submission. However, if you have further questions that help shape your own work, or help build a relationship between you and the stakeholder, you should feel free to ask them.

#### I’m not sure if a sub-office and larger office of the same organization count as separate stakeholders or not. What should I do?

In case of doubt, please complete a form for both, and let me know with your submission that you are not sure. It is better to have too much information rather than not enough. We can then discuss and decide if it is separate or not.
