# Supplementary material for: An applied methodology for stakeholder identification in transdisciplinary research
Source: Sustain Sci. 2016 Jul 26;11(5):763–75. doi: 10.1007/s11625-016-0385-1 (PMC6106094; doi:10.1007/s11625-016-0385-1)
Supplement: Supplementary file 5 — Supplementary material 5 (DOCX 134 kb) [file 11625_2016_385_MOESM5_ESM.docx]

RECARE: Stakeholder Identification

You are being invited to take part in a research project that aims to prevent and remediate degradation of soils in Europe (RECARE). We are currently identifying potential stakeholders so that we can keep them informed about the project (if they wish), and so that we can invite them for participation in the next stages of research. **Today, we would only like to ask you to participate in the stakeholder identification.** Participation in further research will be sought at the appropriate time. Agreeing to participate today does not obligate you to participate in the future. Declining participation today will not prevent your participation in the future if you wish to be involved.

This information sheet will give you more information on the stakeholder identification and your participation in it. Information on the wider RECARE project is provided on the separate project leaflet.

**About the Stakeholder Identification**

The RECARE project recognises that successful land management must be planned and implemented by a broad range of stakeholders, in order to reflect their varying priorities and realities. Therefore, we would like to identify stakeholders in order to understand who plays what roles in land management.

A stakeholder is anyone who can affect, or be affected by, an action or a decision. They may have different interests and act at different scales and some may be hidden. They might be an individual, or an organization or group. They may include farmers, but also land owners, government bodies, extension workers, etc.

In order to identify stakeholders, we are conducting a snowball sample. This means that we have started with a list of possible stakeholders that we are already aware of. We are then contacting a sample of those in order to ask them of other stakeholders that they are aware of. We are also collecting some basic information on what topics and roles they are engaged with (e.g. wildlife protection, crop productivity, etc.).

We are not collecting personal information or any information that may be considered sensitive (e.g. your personal opinions of other stakeholders). We are only collecting information that would be available in the public domain. Your crucial role will be in helping to understand who might be a stakeholder.

**Your Participation**

You have been asked to participate today because we consider you to be a stakeholder in this case study area. Whether or not you agree that you are a stakeholder can be discussed if you wish.

It is up to you whether or not to take part. If you do decide to take part, you will be given this information sheet to keep. You can withdraw at any time without any consequences and you do not have to give a reason. If you withdraw, you can specify whether you would like the information you have provided so far to be removed from the research or not.

If you agree to participate, you will be asked to complete a questionnaire with the help of the researcher present. The questionnaire will ask no personal details beyond your contact information. You do not have to provide your contact information, though it will be useful for keeping you informed of the project and further opportunities to participate. Similarly, if you wish your name not to be used, you can make sure it is not entered on the questionnaire. The questionnaire will ask you information about your possible role in soil management, and about who else you would consider to be a stakeholder.

There are no foreseen risks to participating in this research. However, if you have any concerns, we would be happy to discuss them with you.

**Confidentiality**

Your participation in this stakeholder analysis will be kept confidential. We will not tell anyone that you were part of this identification process, and your name will not appear in any reports (though your organisation will).

You will be asked for your contact details, but this is so that we can keep you informed about the RECARE project and opportunities to participate in the future. You do not have to provide it if you do not want. If you do provide your contact information, it will be kept by the researcher and used only for the purposes of the RECARE project.

If you have any questions about the RECARE project or the stakeholder identification and your role, please ask:

RESEARCHERS TO INSERT THEIR CONTACT DETAILS.

www.recare-project.eu

Thank you for taking the time to read through this information and consider participating in the research project.
